# Jump-GRS: a multi-phase approach to structured pruning of neural networks for neural decoding

**DOI:** 10.1088/1741-2552/ace5dc

**Published:** 2023-07-31

**Authors:** Xiaomin Wu, Da-Ting Lin, Rong Chen, Shuvra S Bhattacharyya

**Affiliations:** 1 Department of Electrical and Computer Engineering, University of Maryland, College Park, MD 20742, United States of America; 2 Department of Diagnostic Radiology and Nuclear Medicine, University of Maryland School of Medicine, Baltimore, MD 21201, United States of America; 3 Institute for Advanced Computer Studies (UMIACS), University of Maryland at College Park, College Park, MD 20742, United States of America; 4 National Institute on Drug Abuse, Gaithersburg, MD 20892, United States of America

**Keywords:** neural decoding, machine learning, neural network pruning

## Abstract

**Objective.:**

Neural decoding, an important area of neural engineering, helps to link neural activity to behavior. Deep neural networks (DNNs), which are becoming increasingly popular in many application fields of machine learning, show promising performance in neural decoding compared to traditional neural decoding methods. Various neural decoding applications, such as brain computer interface applications, require both high decoding accuracy and real-time decoding speed. Pruning methods are used to produce compact DNN models for faster computational speed. Greedy inter-layer order with Random Selection (GRS) is a recently-designed structured pruning method that derives compact DNN models for calcium-imaging-based neural decoding. Although GRS has advantages in terms of detailed structure analysis and consideration of both learned information and model structure during the pruning process, the method is very computationally intensive, and is not feasible when large-scale DNN models need to be pruned within typical constraints on time and computational resources. Large-scale DNN models arise in neural decoding when large numbers of neurons are involved. In this paper, we build on GRS to develop a new structured pruning algorithm called jump GRS (JGRS) that is designed to efficiently compress large-scale DNN models.

**Approach.:**

On top of GRS, JGRS implements a ‘jump mechanism’, which bypasses retraining intermediate models when model accuracy is relatively less sensitive to pruning operations. Design of the jump mechanism is motivated by identifying different phases of the structured pruning process, where retraining can be done infrequently in earlier phases without sacrificing accuracy. The jump mechanism helps to significantly speed up execution of the pruning process and greatly enhance its scalability. We compare the pruning performance and speed of JGRS and GRS with extensive experiments in the context of neural decoding.

**Main results.:**

Our results demonstrate that JGRS provides significantly faster pruning speed compared to GRS, and at the same time, JGRS provides pruned models that are similarly compact as those generated by GRS.

**Significance.:**

In our experiments, we demonstrate that JGRS achieves on average 9%–20% more compressed models compared to GRS with 2–8 times faster speed (less time required for pruning) across four different initial models on a relevant dataset for neural data analysis.

## 1. Introduction

Deep learning [1] is a powerful tool to learn valuable features from input data, and can be used in a great variety of machine learning tasks. Deep learning models are widely applied in different application areas, including computer vision, natural language processing, and neuroscience.

Multi-layer perceptrons (MLPs) [2] and convolutional neural networks (CNNs) [3] are two common types of deep neural network (DNN) models. MLPs and CNNs are widely used in classification and prediction tasks with vector- and matrix-format data. MLPs are composed of consecutive dense layers, which are analogous to biological neural structures. MLPs are effective at extracting input features and are often used in classification tasks. CNNs apply convolution computations and pooling techniques to extract features from input data. CNNs often have multiple dense layers at the end of a network (output side) to summarize the extracted features. CNNs are often used for feature extraction and classification from images.

The size and structure of MLPs and CNNs generally have large influence on the performance of machine learning tasks on datasets containing massive amounts of information. Models with large sizes and complicated structures often require a long time to train and can overfit the training data. Here, by model size we mean the number of weight parameters of the given network. In many cases, models with large sizes eventually converge and give high-quality predictions on validation data [4]. Models with small sizes and simple structures take less time for training and run faster during inference. However, such models may have trouble extracting large portions of useful information from the given datasets and therefore may limit prediction performance.

It is common to construct and use an overparameterized model when the model accuracy is the only consideration. However, when a DNN is applied in a time- or resource constrained application, such as a real-time or Internet-of-things (IoT) application, it is often important to derive a compact DNN model, where the model size is streamlined while maintaining sufficient prediction accuracy [5].

### 1.1. Related work on pruning

Pruning is a general approach to deriving compact models. Pruning involves taking an overparameterized or otherwise less compact model and removing selected weights that are considered to not have significant contribution to the predictive capability of the enclosing network [6]. In this paper, we develop a novel pruning approach that has important applications in neural decoding and in other neural engineering applications that involve DNN-based analysis of large collections of neurons.

DNNs have grown in popularity in the neural decoding field due in part to their potential for high-accuracy decoding [7, 8]. In traditional DNN application areas, such as computer vision and natural language processing, DNN models for large-scale inputs and high-accuracy performance often have very large size [9].

Some works involving DNNs for neural decoding focus on decoding accuracy (e.g. see [10, 11]). However, for neural decoding applications, the speed of inference is also important—for example, a compact DNN model can help to perform real-time neural decoding in the context of closed-loop neuromodulation [12]. Many methods for pruning DNN models have been introduced in the literature (e.g. see [13-17]). These methods aim to reduce DNN model size and increase inference speed while minimizing any degradation in inference accuracy. Among previously-developed pruning methods, NeuroGRS provides an approach that is demonstrated to be particularly well-suited to neural decoding [17]. In the name NeuroGRS, the acronym GRS stands for Greedy inter-layer order with Random Selection (GRS), which is the name of the novel pruning algorithm that is introduced as part of NeuroGRS.

References [16, 18] focus on weight magnitude based pruning. Wu *et al* [17] focuses on detailed model structure search. The works of [16, 18] can achieve fast pruning speed by pruning large portions of minimally-contributing weights before each retraining cycle. The method presented in [17] can reach highly compressed models by sacrificing pruning speed and deriving compact model structures by performing detailed structure search. In this work, we found that the structure-search process of [17] can be optimized to have faster pruning speed while keeping all of its advantages in terms of deriving compact final structures.

In this work, we use two baselines for our experimental comparisons. First, we use Natural inter-layer order Weight Magnitude based intra-layer pruning (NWM), which encompasses the structured pruning approaches used in [16, 18]. Our second baseline is Greedy inter-layer order Random intra-layer Selection pruning (GRS), which represents the approach presented in [17]. We conducted extensive experiments to show that our proposed new method, jump GRS (JGRS), can reach faster pruning speeds than GRS, while also deriving more compact models compared to NWM.

### 1.2. Related work: NeuroGRS

NeuroGRS is a software tool that is geared towards pruning DNN models, such as MLP or CNN models, for neural decoding applications [17]. DNN models often contain weights that have little or no significance, and without which a DNN model can still achieve similar accuracy performance. A pruned DNN model generally allows faster training and inference compared to the original overparameterized neural network. A pruned DNN can also be significantly more interpretable than its original form. This higher degree of interpretability can help neuroscientists analyze relationships between specific neurons and behavior prediction decisions produced by neural decoding [19].

NeuroGRS [17] and this work focus on a particular form of pruning called *structured pruning*, which involves the removal of regular patterns of connections from the given network instead of allowing removal from arbitrary points in the network. For more background on structured pruning, we refer the reader to [20]. Results from structured pruning can be exploited in DNN implementations without any need for specialized hardware or software libraries. Without specialized hardware or software support, unstructured pruning approaches may increase the computational complexity of the network, thereby having the opposite of the effect that is usually desired from pruning.

NeuroGRS [17] introduces a new structured pruning algorithm, called GRS, and combines GRS with two state-of-the-art unstructured pruning methods [16, 21] together into a unified software tool. The unstructured pruning methods within NeuroGRS can be applied optionally if appropriate hardware support or software libraries are available to exploit the corresponding pruning results. NeuroGRS can prune an overparameterized DNN model into a compact form. The resulting compact form, compared to the original model, has faster training and inference speed and a smaller model size, while providing comparable classification performance in terms of accuracy.

GRS is different from other structured pruning methods in its joint consideration of both trained weights and model structure when generating compact models. However, one limitation is its computationally intensive nature, which makes it unsuitable for large DNNs, such as those arising in the analysis of neural signals from large collections of neurons.

### 1.3. Advantage of compact models

Compact DNNs are useful for real-time neural decoding tasks, such as brain computer interfaces [22] and precise neuromodulation [23]. Compact models can reduce the time used for model inference and possibly allow additional runtime to be devoted to other important preprocessing tasks, such as neuron detection and motion correction [24]. In other words, the efficiency of compact models can help to achieve real-time performance if execution speed is a critical factor, or to improve the effectiveness of other system-tasks if there is some slack in achieving real-time performance.

Recent advances in calcium imaging can observe up to a million neurons. Large DNN models are needed to effectively extract information from large-scale neural signals. Inference of such large-size DNN models is very challenging to perform in real-time without significant reduction in model size. Thus, compact DNN models provide the potential to make better use of state-of-the-art calcium imaging technology for the acquisition of neural signals.

### 1.4. Contribution of JGRS

In this paper, we build on the methods in GRS for joint weight/structure consideration, while developing new techniques to reduce time complexity, and allow for scaling up to large DNNs. We refer to our proposed new pruning algorithm as JGRS. JGRS also incorporates parameterization to allow the neural decoding system designer to systematically explore trade-offs among computational efficiency, accuracy impact, and reduction in network complexity (pruning effectiveness) in the pruning process. For example, during system prototyping, the designer may prefer a pruning process that favors computational efficiency (fast pruning), whereas when the system is being finalized for deployment, more emphasis may be placed on reducing the complexity of the pruned network.

JGRS was developed through careful analysis of the operation of GRS. Through this analysis, which included experimenting with GRS and studying intermediate pruning structures produced by the algorithm, we observed that GRS often operates through two distinct phases of operation, which we refer to as the *far phase* and the *near phase*. By an *intermediate structure* during the pruning process of GRS, we mean the network that results from the first *i* channel removals performed by GRS, where *i* is some integer that is less than the total number of channel removals performed on the given network. In the *far phase*, intermediate structures are relatively far (different) from the final compact form derived by the algorithm; instead, the intermediate structures are closer to the overparameterized initial network. On the other hand, in the near phase, intermediate structures are closer to the final form.

In our analysis of GRS operation, we also observed that the model being pruned is generally more sensitive to structural changes in the *near phase* compared to the *far phase*. Here, by *more sensitive*, we mean that model performance in terms of accuracy tends to be affected more heavily by changes in model structure. We introduce a technique, called the *jump* mechanism in JGRS, during the far phase that exploits the relative insensitivity during this phase to structural changes. Intuitively, the jump mechanism avoids repeated retraining and validation iterations, thereby allowing successive pruning operations to be carried out much more quickly than in the original GRS algorithm. More details on the jump mechanism are presented in section 2.

JGRS can be viewed as a modified version GRS, where the pruning process is decomposed into far and near phases, as described above, and the jump mechanism is integrated into the far phase to significantly accelerate the overall operation of the pruning process. We perform extensive experiments to demonstrate that JGRS provides large reductions in pruning time compared to GRS without significant impact on the prediction accuracy of the final, compact networks that are produced. In addition to presenting the JGRS algorithm and its extensive experimental evaluation, this paper studies the two-phase phenomena associated with the near and far phases, and provides useful insight that has more general implications to network pruning (beyond development of the JGRS algorithm).

The main contributions of this work can be summarized as follows.

- We identify and study a multi-phase phenomenon in structured pruning and analyze weaknesses of GRS under this multi-phase phenomenon.
- We introduce a ‘jump mechanism’, as described in section 2.2, to help reduce unnecessary structure search in GRS, and improve the pruning speed of GRS significantly without compromising the model-compression ability of the original GRS.
- We conduct extensive experiments to demonstrate the identified multi-phase phenomenon, and we show that JGRS outperforms GRS in both compression ability and pruning speed. Moreover, we show that JGRS derives more compact pruned models compared to other neural network pruning methods in the literature.

## 2. Methods

In this section, we first analyze important characteristics involved in the operation of the GRS algorithm. Next, based on this analysis, we present a significant modification to GRS that helps to retain the key advantages of GRS while addressing its key weakness, which is its long runtime, and an associated lack of scalability to large DNN models.

### 2.1. Analysis of the GRS algorithm

NeuroGRS and the underlying GRS algorithm are developed to generate compact DNN models that accelerate inference speed by eliminating selected weights while maintaining model accuracy within an acceptable range. GRS exhaustively examines intermediate sub-structures of the input overparameterized neural network. Units are selected as candidates in a randomized manner, and selection among candidates is performed using a greedy strategy. The exhaustive assessment evaluates all possible pruning results in the current step and uses a greedy strategy to select the best result of the current step to carry over into the next iteration. More details about how the GRS algorithm can be found in [17].

An advantage of such a retraining-intensive approach in GRS is that the approach can accurately assess the accuracy impact of each pruning candidate with a diverse range of candidates considered at each pruning iteration. However, there is a disadvantage in that retraining in each iteration costs a large amount of computational power. As demonstrated in [17], such an investment of computational effort can be beneficial for certain types of information extraction from neural signals where relatively small neural networks are involved. However, the scalability of the GRS technique to large networks is limited.

In our extensive experimentation with GRS, we have observed an interesting pattern in which the pruning process often progresses through two distinct phases of operation. We refer to the first of the regimes as the *far phase* because during this phase, the intermediate pruned models are relatively far from their final compact form. Similarly, we refer to the second phase, where the model is approaching its final form, as the *near phase*. This phenomenon is demonstrated concretely in section 3.3. Based on these experimental observations, which we have observed consistently, we hypothesize that optimizing the accuracy/efficiency trade-off of pruning will benefit from significantly different approaches in these two phases.

To intuitively describe the hypothesized multi-phase phenomenon in the pruning process, we consider a multi-layer-perceptron (MLP) network as an example. Suppose an MLP model has more than enough representation power with respect to the given accuracy requirement—that is, the model has sufficient connections between successive layers. Intuitively, in that case, a learned informative forward path *P* can be transferred to other nodes in the model through a sufficient amount of retraining. However, if an MLP model is in a relatively compact form, then the informative path *P* may be impossible to retain if some critical node is removed. The reduced sensitivity to specific pruning operations in the higher-connectivity case (far phase) opens up the possibility for more computationally efficient strategies. More intensive computational effort can then be devoted to the near phase, a heightened-sensitivity phase, where the effort can be utilized more productively towards the ultimate goal of accuracy-constrained compactness optimization.

### 2.2. JGRS

We formulate the JGRS algorithm as a structured pruning algorithm that can be applied to CNNs or MLPs. Extension to other classes of DNNs is an interesting direction for future work. As a structured pruning approach, JGRS removes relatively coarse-grained components from the input DNN (CNN or MLP) as its basic unit of removal. In particular, it removes filters and nodes for CNNs and MLPs, respectively, as its basic unit of removal. Each removal operation in the algorithm involves either a whole filter or a whole node. Henceforth, we refer to these basic units of removal as *removal units* in our discussion of JGRS.

As the JGRS algorithm operates on an input DNN model M, it generates a sequence $I_{0},I_{1},I_{2},\ldots ,I_{n}$ of progressively more compact DNNs, where $I_{0}=M$, $I_{n}$ is the final compact DNN produced by the algorithm, and for each $i=1,2,\ldots ,n,\;I_{i}$ is derived from $I_{i-1}$ by removing one or more removal units from $I_{i-1}$. We refer to $I_{0}$ as the input to JGRS, $I_{1},I_{2},\ldots ,I_{n-1}$ as the intermediate models generated by JGRS when applied to $I_{0}$, and $I_{n}$ as the output produced by JGRS corresponding to the input $I_{0}$. Since JGRS employs randomization techniques, the output $I_{n}$ is not unique for a given $I_{0}$; the output in general depends on the mechanism used to guide randomized decisions, and how the mechanism is initialized (seeded).

We have designed the JGRS algorithm such that it retains GRS as a core pruning approach, but incorporates along with the approach to exploit the insights described in section 2.1 about far and near phases in the operation of GRS. The design of the algorithm is broken down into far and near phases of operation, where different strategies are used in each phase. The definitions of the far and near phases for the JGRS algorithm are intended to capture the corresponding phases of GRS algorithm operation that are described in section 2.1.

We define the *compression rate*, denoted *CR*, as the number of removal units that are eliminated from the network between successive retraining and validation operations. Intuitively, a higher *CR* leads to faster pruning but has more risk of reducing model accuracy. Based on the discussion in section 2.1, this risk is much lower during the far phase compared to the near phase.

In addition to the model M that is to be pruned, another important input to JGRS is the minimum unit-count vector (MUCV), denoted μ. The vector μ is a positive-integer vector that is indexed by the hidden layers in M. For each hidden layer λ, μ[λ] specifies a constraint on the minimum number of units (removal units) in λ that must be retained by the pruned solution computed by JGRS. The vector μ thus gives the neural decoding system designer a mechanism to control the maximum degree of pruning that will occur in each layer. Moreover, experimentation with different settings of μ gives the system designer a means for exploring accuracy/compactness trade-offs associated with a given prediction framework for neural decoding. Investigation into automated mechanisms for configuring μ is an interesting direction for future work.

Given a DNN M, an MUCV μ, and a hidden layer λ in M, we define the *pruning midpoint*, denoted midpoint(λ), by:

(1) $$midpoint(\lambda )\;\;=max\left(floor\left(\frac{unitCount(M,\lambda )-\mu (\lambda )}{2}\right),1\right),$$

where unitCount(M,λ) denotes the number of removal units contained in hidden layer λ within M.

The far phase of JGRS is decomposed into two sub-phases, called *far subphase 1* and *far subphase 2*. In far subphase 1, midpoint(λ) removal units are removed from each hidden layer before retraining and validation are invoked. Thus, the *CR* in far phase 1 can be expressed as: $\sum_{\lambda =1}^{N_{H}}midpoint(\lambda )$, where $N_{H}$ denotes the number of hidden layers in the given DNN.

On the other hand, in far subphase 2, retraining and validation are invoked after each hidden layer λ is processed, where the processing in this context involves removing midpoint(λ) removal units. Thus, the *CR* in far subphase 2 depends on the hidden layer that is being processed, and can be expressed simply as midpoint(λ).

In contrast to the two far subphases, where retraining and validation steps are interleaved with batches of pruning operations, the near phase performs retraining and validation after elimination of each selected removal unit. The *CR* is therefore reduced to 1 in the near phase. This is because of the significantly heightened sensitivity of accuracy to pruning that is characteristic of the near phase.

Table 1 summarizes the compression rates in the successive stages through which JGRS operates.

As JGRS progresses through the pruning process, it progressively decreases the *CR*—that is, it progressively increases the frequency of retraining and validation steps. The bypassing of retraining and validation accelerates operation of the pruning process. The reduced sensitivity in the far phase of accuracy to pruning operations allows us to perform this bypassing without significantly degrading the model’s predictive capability. The jump mechanism, which was introduced in section 1 and is the basis for the name ‘JGRS’, is based on this bypassing of retraining and validation. In the far phase, JGRS effectively ‘jumps across’ groups of removal unit eliminations (pruning operations) before it re-invokes retraining and validation. On the other hand, there is no jumping in the near phase, where retraining and validation are interleaved with individual pruning operations.

The speedup of JGRS is achieved by greatly reducing the number of times that training and validation need to be performed throughout the pruning process. In GRS, training and validation are performed after the deletion of each removal unit throughout the entire pruning process. In JGRS, such frequent invocation of training and validation occurs only during the near phase. In far subphase 2, training and validation occurs much less frequently compared to the near phase, and in far subphase 1 training and validation are almost entirely bypassed. In our experiments, we have observed that among all of the removal units that are deleted during the JGRS pruning process, only a small portion of the deletions occur during the near phase. The other deletions occur with a much more streamlined integration of training and validation compared to what is used in GRS.

**Table:** 

Algorithm 1: *far_subphase_*1 function: a pseudocode sketch of the portion of JGRS that operates during far subphase 1.
Input:M:input DNN model.DT:training dataset.DV:validation dataset.μ:minimum unit-count vector.ValAccmin:minimum validation accuracy.Output:MFS1:pruned DNN model that approximatesM.Acc:accuracy ofMFS1.NFLOPs:the number of FLOPs inMFS1.Nparams:the number of parameters inMFS1.OriValAcc←validation(M,DV)Mstart,IMS←MlastValAcc←OriValAccValAcc←OriValAccwhileValAcc>=ValAccmindo∣minStructReached←TrueforeachλinMstart.hiddenLayersdo∣ifλ.UnitCount>μ[λ]then∣minStructReached←FalseunitDistance←max(floor((λ.UniCount−μ[λ]∕2),1)IMS←randomCutUnits(IMS,λ,unitDistance)∣∣endifminStructReachedthen∣ValAcc←0else∣fineTuning(IMS,DT)ValAcc←validation(IMS,DV)∣ifValAcc>=ValAccminthen∣lastValAcc←ValAccMstart←IMS∣∣endMFS1←MstartAcc←lastValAccNFLOPs←FLOPCount(MFS1)Nparams←ParamCount(MFS1)returnMFS1,Acc,NFLOPs,Nparams

Algorithm 1 provides a pseudocode sketch of the portion of JGRS that operates during far phase 1. We refer to this portion of JGRS as the *far_subphase_*1 function. This function takes five arguments: the input DNN model M that is to be pruned, a training dataset $D_{T}$, a validation dataset $D_{V}$, an MUCV μ, and a constraint on the validation accuracy *ValAcc_min*. The argument *ValAcc_min* gives the minimum accuracy, as determined by evaluation on $D_{V}$, that is acceptable by a solution produced by *far_subphase*_1.

The function *validation(m, d)* in Algorithm 1 evaluates the input model m on the input dataset d and returns the resulting prediction accuracy. The function *randomCutUnits*(*m, l, num_units*) takes as argument a DNN model m, a layer l within m, and a positive integer *num_units*. The function randomly removes *num_units* removal units from the layer l of the model m and returns the modified DNN model. The function *fineTuning*(*m, d*) retrains the model m with the input dataset *d.* The functions *FLOPCount*(*m*) and *ParamCount*(*m*) return the number of floating-point operations (FLOPs) and parameters, respectively, in the given model *m.* In Algorithm 1, IMS stands for ‘intermediate model structure’,

Algorithm 2 provides a pseudocode sketch of the *far_subphase*_2 function, which is the portion of JGRS that operates during far phase 2. The *validation, randomCutUnits*, and *fineTuning* functions are the same as the corresponding functions that are called in Algorithm 1.

**Table:** 

Algorithm 2: *far_subphase_*2 Function: A pseudocode sketch of the portion of JGRS that operates during far subphase 2.
Input:M:input DNN model.DT:training dataset.DV:validation dataset.μ:minimum unit-count vector.ValAccmin:minimum validation accuracy.Output:MFS2:pruned DNN model that approximatesM.Acc:accuracy ofMFS2.NFLOPs:the number of FLOPs inMFS2.Nparams:the number of parameters inMFS2.OriValAcc←validation(M,DV)Mstart,IMS←MGRSlastValAcc←OriValAccmaxValAcc←OriValAccwhilemaxValAcc>=ValAccmindo∣ValAcc←[]IntermediateStructures←[]foreachλinMstart.hiddenLayersdo∣ifλ.UnitCount>μ[λ]then∣unitDistance←max(floor((λ.UniCount−μ[λ])∕2),1)IMS←randomCutUnits(Mstart,λ,unitDistance)fineTuning(IMS,DT)ValAcc.append(validation(IMS,DV))IntermdiateStructures.append(IMS)∣∣endifValAcc==[]then∣maxValAcc←0else∣maxValAcc=max(ValAccs)ifmaxValAcc>=ValAccminthe∣lastValAcc←maxValAccindex←argmax(ValAccs)Mstart←IntermediateStructure[index]∣∣endMFS2←MstartAcc←lastValAccNFLOPs←FLOPCount(MFS2)Nparams←ParamCount(MFS2)returnMFS2,Acc,NFLOPs,Nparams

The overall JGRS algorithm is shown in Algorithm 3. In addition to the original DNN model M, the training and validation datasets $D_{T}$ and $D_{V}$, the MUCV μ, and an accuracy drop tolerance 𝒯, the JGRS algorithm takes three inputs that control the different algorithm stages: attempt_stage_1, attempt_stage_2, and attempt_stage_3. These latter three input arguments control how many attempts are carried out for each pruning stage, where the three successive stages correspond to far phase 1, far phase 2, and the near phase, respectively. By having multiple attempts during each stage, we exploit the randomization inherent in the stage to examine different sets of removal units to eliminate. Using multiple attempts in this manner also helps to reduce bias caused by the underlying randomness.

The accuracy drop tolerance 𝒯 can be expressed as

(2) $$𝒯=\frac{{ValAcc}_{min}}{OriValAcc},$$

where ${ValAcc}_{min}$ is the minimum tolerable accuracy after pruning and OriValAcc is the accuracy of the input model M. The accuracy values in this context are interpreted in relation to the validation dataset $D_{V}$.

In our experiments, we set

(3) attempt_stage_1=attempt_stage_2=attempt_stage_3=3.

The *validation* function in Algorithm 3 is the same as that called in algorithms 1 and 2.

**Figure 1.**
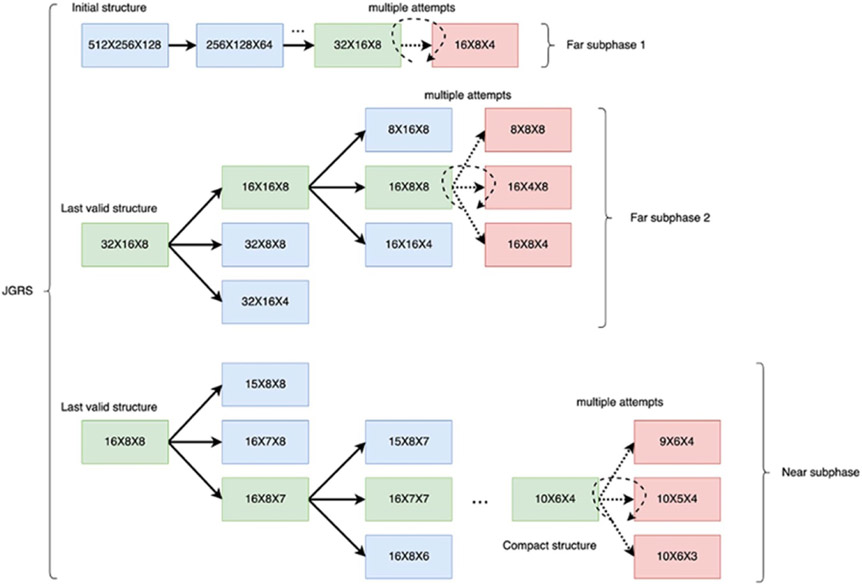
Illustration of JGRS method.

Figure 1 shows an illustration of how JGRS operates in different phases with an example initial model structure.

**Table:** 

Algorithm 3: A pseudocode sketch of the *JGRS* algorithm.
Input:M:input DNN model.DT:training dataset.DV:validation dataset.μ:minimum unit-count vector.T:tolerance of accuracy drop.attempt_stage_1:the amount of attempts forfar_subphase_1.attempt_stage_2:the amount of attempts forfar_subphase_2.attempt_stage_3:the amount of attempts for the GRS pruning.Output:MJGRS:pruned DNN model that approximatesM.Acc:accuracy ofMJGRS.NFLOPs:the number of FLOPs inMJGRS.Nparams: the number of parameters inMJGRS.Acc←validation(M,DV)ValAccmin←Acc×TNFLOPs←FLOPCount(M)Nparams←ParamCount(M)MJGRS←Mwhileattempt_stage_1>0do∣attempt_stage_1−=1MJGRS,Acc,NFLOPs,Nparams=far_subphase_1(MJGRS,DT,DV,μ,ValAccmin)∣endwhileattempt_stage_2>0do∣attempt_stage_2−=1MJGRS,Acc,NFLOPs,Nparams=far_subphase_2(MJGRS,DT,DV,μ,ValAccmin)∣endwhileattempt_stage_3>0do∣attempt_stage_3−=1MJGRS,Acc,NFLOPs,Nparams=GRS(MJGRS,DT,DV,μ,ValAccmin)∣endreturnMJGRS,Acc,NFLOPs,Nparams

## 3. Experimental results

In this section, we present an extensive experimental evaluation, which concretely demonstrates the effectiveness of JGRS in the context of calcium-imaging-based neural decoding.

### 3.1. Dataset

Our experiments involve a collection of datasets called MSN (medium spiny neurons). MSN is a collection of datasets involving calcium imaging sessions with mouse models; details about this dataset collection can be found in [25]. In our experiments, we use the 9 datasets in MSN from the first three imaging sessions (e1, e2, e3) of subjects 04, 05, 06; these are the same datasets that GRS has been evaluated on in [17], and therefore they provide a natural testbed for evaluating JGRS. Furthermore, to provide more extensive evaluation, we use 9 additional datasets from MSN—these are from the first three imaging sessions (e1, e2, e3) with three different subjects, 07, 08, 09.

Each of the 18 datasets that we worked with contains a 3000×β(T) matrix Z(T) of floating point values for each mouse T, and a 3000-element vector L(T) of binary values. Here, β(T) denotes the number of detected neurons associated with T. The rows of Z(T) and elements of L(T) correspond to distinct sampling intervals, which are spaced 0.1 seconds apart (due to the 10 Hz sampling rate) and collectively cover 5 minutes of imaging activity. Each column of Z(T) corresponds to a distinct neuron. Each matrix element Z(T)[i,j] gives the value of the measured calcium imaging sample for neuron j at sample index i. Similarly, each element L(T)[i] gives binary a label indicating whether or not the mouse was engaged in fine motion at the time instant associated with sample index i. Fine motion at a given time instant t means that the mouse is moving at a speed s(t) such that ${0.2\;\text{cm}\;s}^{-1}<s(t)<{2\;\text{cm}\;s}^{-1}$). The mouse models whose data we used in our experiments, models 04, 05, 06, 07, 08, 09, have β(T)=273,140,114,154,53,96 neurons, respectively.

Another dataset that we used in our experiments consists of embedding vectors of functional microcircuits generated by the Weighted Graph Embedding with Vertex Identity Awareness (WGEVIA) algorithm [26]. These embedding vectors were generated when WGEVIA was applied to a functional microcircuit dataset called REAL [27]. The REAL dataset is processed from a reward zone study in [27]. Animals involved in this study were approved by the Columbia University Institutional Animal Care and Ethics Committee. In this study, a mouse received water rewards when entering and licking a fixed reward zone on a treadmill belt. We refer to this dataset as the *WGEVIA-REAL Embedding Vector Dataset* or simply the *WGEVIA-REAL* dataset for short. Functional microcircuits are collections of neurons that are spatially close to one another and exhibit some form of synchrony in their associated neural activities. Functional microcircuit can be measured by calcium imaging. Functional microcircuits can be represented by graphs in which vertices correspond to neurons and edges represent pairs of neurons that are related in terms of spatial proximity and synchrony. WGEVIA is an algorithm for transforming graphical models, such as those used for microcircuits, into low-dimensional vector representations, called graph-level *embedding vectors*, that are effective for processing by DNNs. Each embedding vector in the WGEVIA-REAL dataset is an 80 dimensional vector associated with a binary label indicating one of two categories of the original functional microcircuit. The dataset contains 1600 embedding vectors evenly labeled with ‘1’ and ‘0’ labels. More details about the WGEVIA-REAL dataset can be found in [26].

The train, validation, and testing samples are randomly taken from the total samples with a 8:1:1 ratio.

### 3.2. Input models

**Figure 2.**
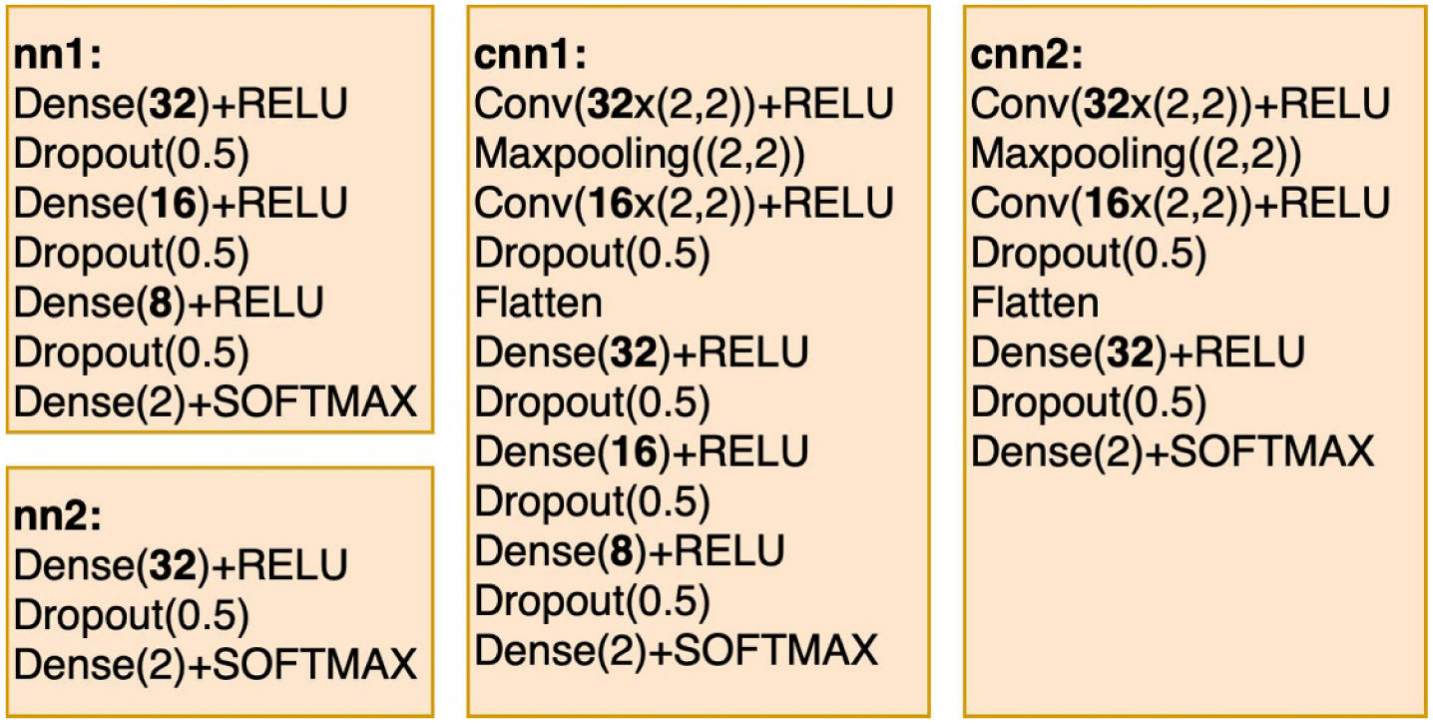
Overparameterized DNN models that are used as input for pruning in our experiments.

**Figure 3.**
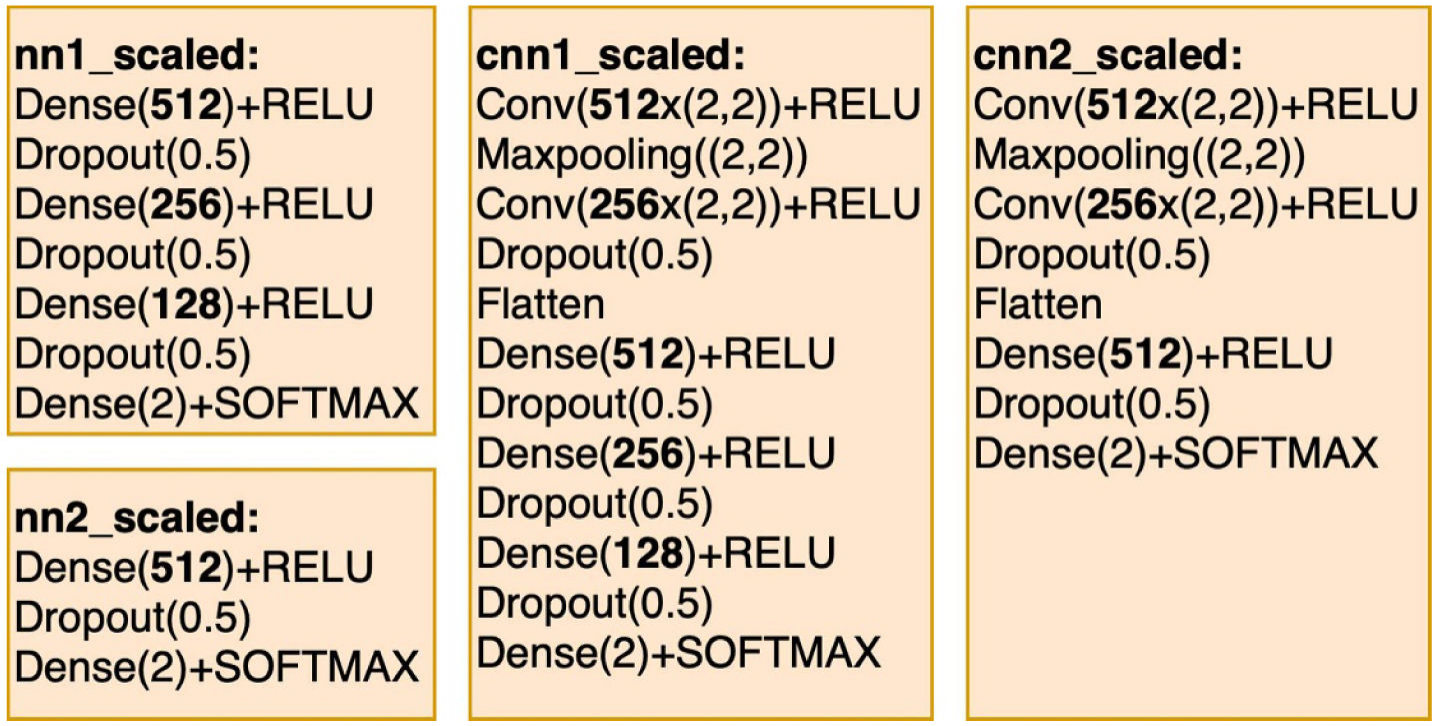
Scaled DNN models that are used to exercise the ability of JGRS to handle different kinds of DNN structures.

Our experiments involve several overparameterized models that are used as input for pruning. These models are summarized in figures 2 and 3. Two different types of DNNs are included in figure 2: two of the models summarized are MLP models—nn1 and nn2—and other two models, cnn1 and cnn2, are CNNs. The models summarized in figure 2 are the same overparameterized models that are used the experiments reported in [17] for the GRS algorithm. We use the models nn1, nn2, cnn1 and cnn2 mainly to compare the pruning performance of JGRS with GRS.

The four scaled models shown in figure 3 hold the same numbers of layers compared to corresponding models in figure 2 but contain 16 times more artificial nodes in each hidden layer. These four scaled models are used to experiment with the pruning performance when applying JGRS on large input models. The purpose of having initial models with different network types, numbers of layers, and numbers of hidden nodes is to test the ability of JGRS to handle different types of DNN structures.

### 3.3. Multi-phase behavior in GRS

In this experiment, we show that as removal units are removed from a DNN, the model being pruned becomes increasingly sensitive to the elimination of removal units as the model becomes more compact. This experiment uses the 9 MSN datasets related to mouse models 04, 05, 06.

For this experiment, we use the Random inter-layer order and Random Selection of intra-layer unit (RRS) algorithm [17], which selects intermediate structures for pruning randomly rather than using the greedy strategy of GRS. We use RRS in this experiment because our objective is to demonstrate the sensitivity of accuracy to pruning as a model becomes more compact rather than to demonstrate specific characteristics of GRS or its underlying greedy strategy. The accuracy-drop tolerance 𝒯 in RRS is set to 0.5 to help ensure that RRS pruning reaches the preset MUCV μ.

As input, we take a 16 × 16 × 16 multi-layer MLP model *mlpmulti* with 16 nodes in each hidden layer. One purpose of selecting this hidden structure is to avoid unbalanced substructures, where only one layer does not get pruned to the number of artificial nodes defined in μ. The MUCV μ is set as 2 × 2 × 2. We run RRS with the *mlpmulti* model on the 9 MSN datasets and observe the pruning trend. We plot the average of the validation accuracy levels of all of the intermediate structures derived from removing a single artificial node from each layer. The RRS iterates with the derived intermediate structures, each having progressively smaller size, until the MUCV μ is achieved. For each intermediate structure evaluated through validation as the RRS algorithm progresses, we averaged across three evaluations to reduce bias due to randomness in the retraining process.

**Figure 4.**
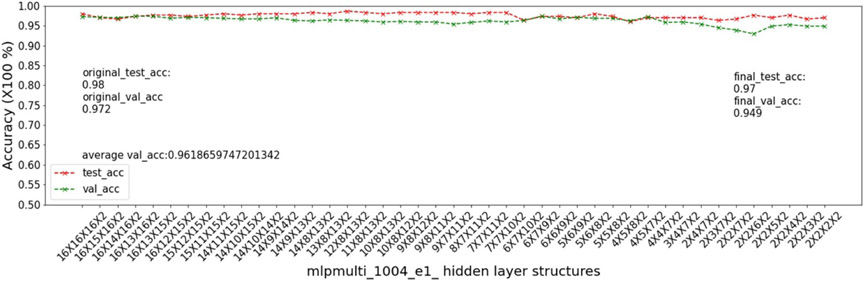
Trends of validation accuracy and test accuracy when pruning an over-parameterized MLP model with hidden structure 16×16×16 to the preset minimum hidden structure 2×2×2 on dataset 04e1.

**Figure 5.**
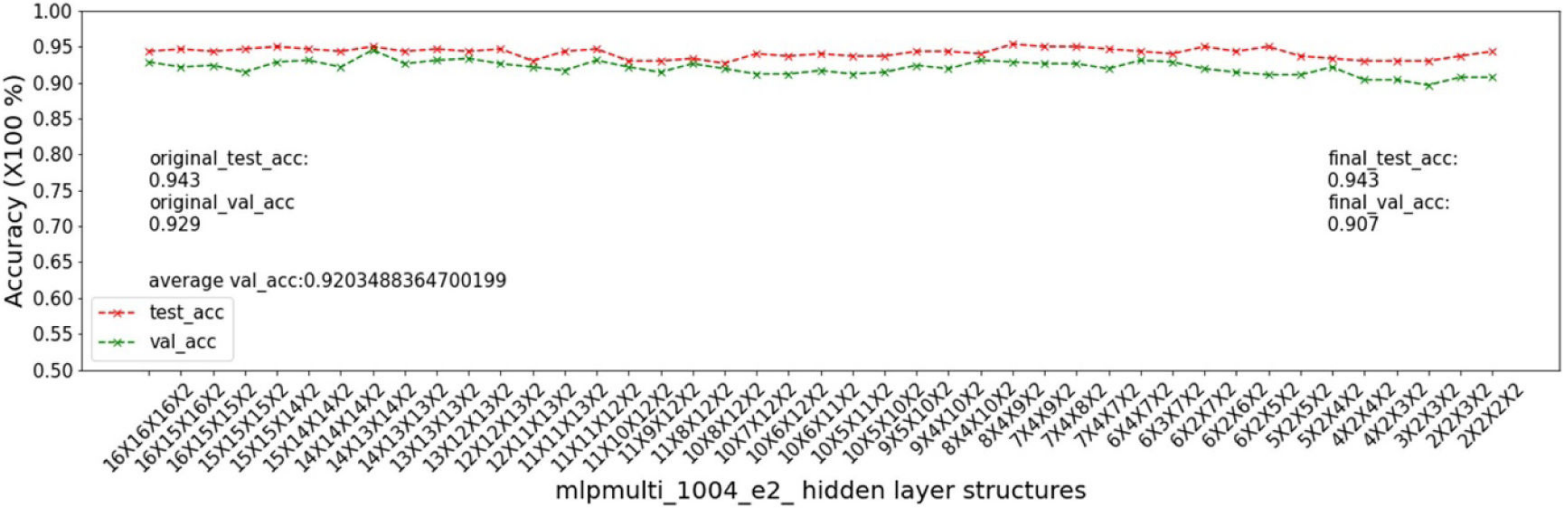
Trends of validation accuracy and test accuracy when pruning an over-parameterized MLP model with hidden structure 16×16×16 to the preset minimum hidden structure 2×2×2 on dataset 04e2.

**Figure 6.**
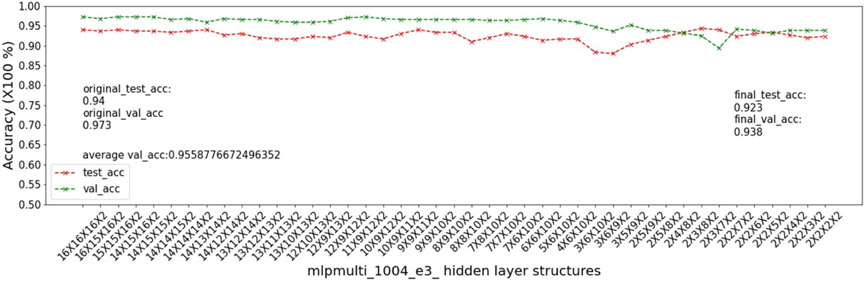
Trends of validation accuracy and test accuracy when pruning an over-parameterized MLP model with hidden structure 16×16×16 to the preset minimum hidden structure 2×2×2 on dataset 04e3.

**Figure 7.**
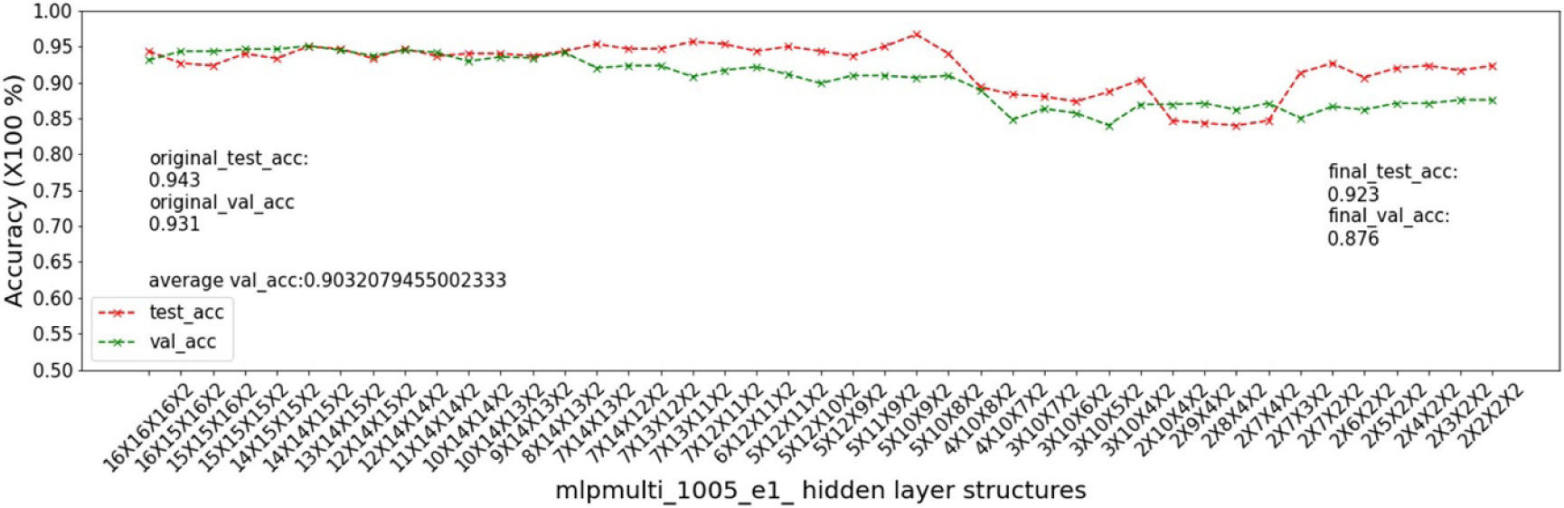
Trends of validation accuracy and test accuracy when pruning an over-parameterized MLP model with hidden structure 16×16×16 to the preset minimum hidden structure 2×2×2 on dataset 05e1.

**Figure 8.**
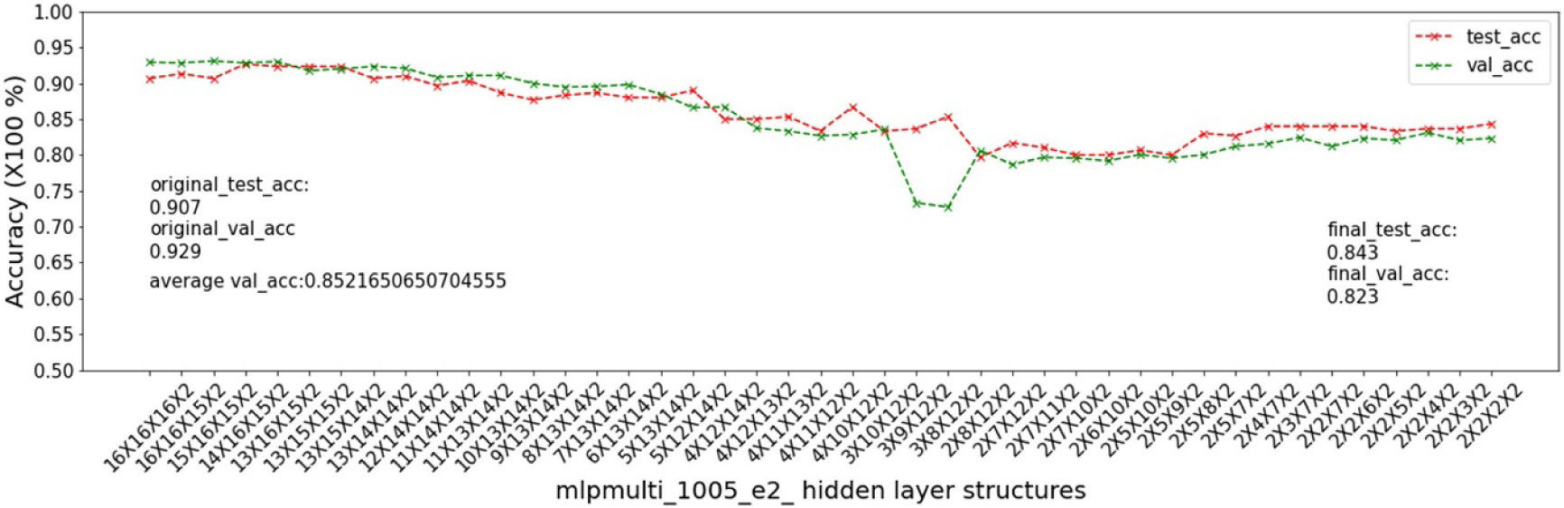
Trends of validation accuracy and test accuracy when pruning an over-parameterized MLP model with hidden structure 16×16×16 to the preset minimum hidden structure 2×2×2 on dataset 05e2.

**Figure 9.**
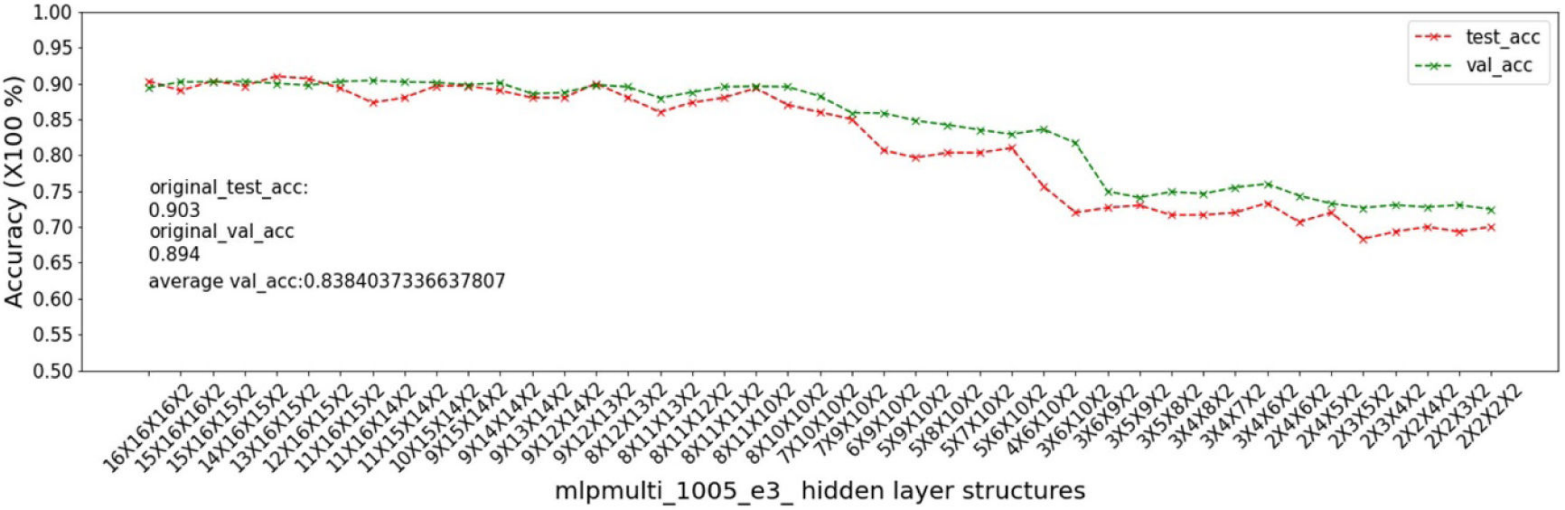
Trends of validation accuracy and test accuracy when pruning an over-parameterized MLP model with hidden structure 16×16×16 to the preset minimum hidden structure 2×2×2 on dataset 05e3.

**Figure 10.**
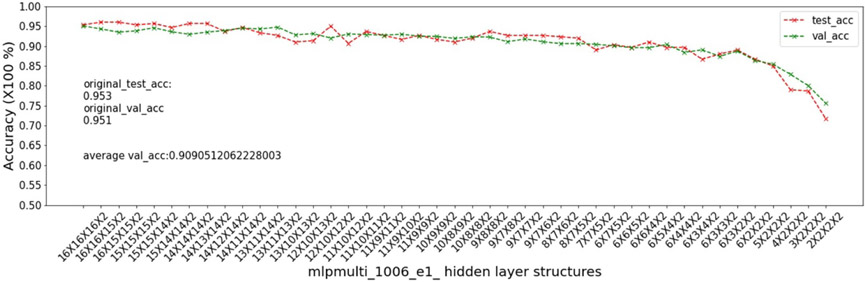
Trends of validation accuracy and test accuracy when pruning an over-parameterized MLP model with hidden structure 16×16×16 to the preset minimum hidden structure 2×2×2 on dataset 06e1.

**Figure 11.**
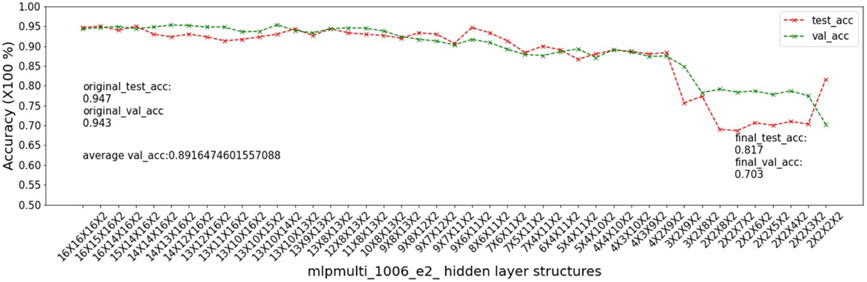
Trends of validation accuracy and test accuracy when pruning an over-parameterized MLP model with hidden structure 16×16×16 to the preset minimum hidden structure 2×2×2 on dataset 06e2.

**Figure 12.**
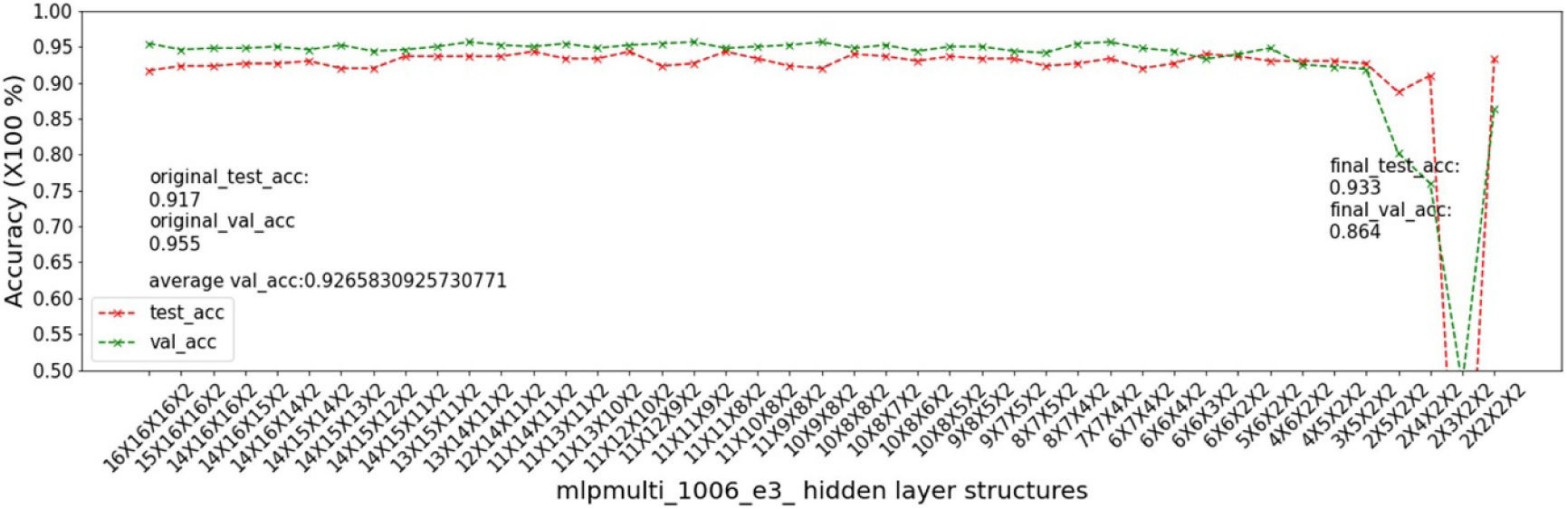
Trends of validation accuracy and test accuracy when pruning an over-parameterized MLP model with hidden structure 16×16×16 to the preset minimum hidden structure 2×2×2 on dataset 06e3.

We show the results of all 9 datasets in figures 4-12. In each of these figures, we see that the average validation accuracy across all substructures, plotted in green color, is stable when the model is close to the over-parameterized side—i.e. the left side of each plot. However, the average validation accuracy drops significantly when the model is close to structures that match the MUCV—i.e. the right side of each plot. With GRS, the compact model will generally be found between an intermediate structure at which the average validation accuracy starts to drop significantly and a structure corresponding to the MUCV.

The experimental results presented in figures 4-12 illustrate in detail and with great consistency the phenomena of far and near phases, which was introduced in section 1 and applied in the development of the JGRS algorithm. These results demonstrate more concretely the motivation for using JGRS and why the far_subphase_1 and far_subphase_2 functions can be applied before the original GRS pruning method to derive a much more efficient structured pruning algorithm compared to applying GRS uniformly during both phases.

### 3.4. Comparison between JGRS and GRS

In this section, we experiment with JGRS and GRS on the same MSN datasets to compare their pruning performance, including pruning speed, final compact model size, and test accuracy drop from pruning.

We applied GRS and JGRS to 4 DNN models with different structures and 9 datasets related to subjects 04,05,06. Furthermore, we extended the experiment to 9 new MSN datasets (beyond the datasets used in [17]). These datasets are related to subjects 07,08,09.

To maintain a fair comparison with JGRS, the GRS method used in this experiment is also averaged across three attempts. This averaging is performed to reduce bias from randomness associated with evaluating different randomly-cut nodes. The accuracy drop tolerance is set as 𝒯=0.985 for both JGRS and GRS.

For measurement of pruning speed, we measure the wall time of both methods in an environment with 6 experiment tasks running in parallel. Each experiment task repeats 10 trials on datasets associated with a single subject—for example, datasets the m04e1, m04e2, and m04e3 datasets associated with mouse m04. Results are averaged across the 10 trials. Four input DNN models of different types and structures are pruned in each trial with GRS or JGRS.

We applied the metrics defined in [17] for comparing pruning methods: AL, FCI, and PCI, which stand, respectively, for Accuracy Loss, FLOP Count Improvement, and Parameter Count Improvement. Differences in AL are reported as the maximum, minimum, and average of equation (4):

(4) {∣Acc_G(d)−Acc_J(d)Acc_O(d).∣d∈D}.

Here, d∈D, where D={m04e1,m04e2,…,m09e3} denotes our set of 18 MSN datasets, and Acc_X(d) represents the accuracy measured in our experiments by method X on dataset d for the given input model M. Here, X=O denotes the original model (without any pruning), while X=G and X=J denote the pruned models derived by applying GRS and JGRS, respectively, to the original model. The initial models before pruning for both JGRS and GRS are trained separately for different datasets.

The FCI difference and PCI difference values reported in table 2 are calculated by taking equation (4) and replacing each Acc_X(d) term with FLOPs_X(d) or Params_X(d), respectively, for the corresponding model specifier X.

Intuitively, from the formulation of AL, FIC, and PCI differences, as described above, a positive value for the AL difference in table 2 means that JGRS performs worse than GRS, while positive values for FCI and PCI indicate that JGRS performs better than GRS.

As shown in equation (5), the *Time_ratio* reports the maximum, minimum, and average runtime comparison between J:JGRS and G:GRS for each dataset d. A value higher than 1 indicates that JGRS is faster than GRS, and a value lower than 1 indicates that JGRS is slower than GRS.


(5) {∣Runtime_G(d)Runtime_J(d)∣d∈D}.

From the results shown in table 2, JGRS can reach compact models with around 10% better FCI and PCI than GRS with negligible AL difference. We anticipate that the improved pruning results of JGRS are because JGRS jumps through less important pruning decisions during the *far phase* and examines more critical pruning decisions by evaluating different candidate-sets of nodes for removal. In addition to improved FCI and PCI, JGRS achieves significantly lower pruning time than GRS when processing models with the same over-parameterized structures.

The minimum observations (results in the column labeled ‘min’) show trends that are opposite to the general trends described above. These deviations from the general trends arise due to randomness in the 10 trials for each model on 18 sub-datasets of MSN. To explain this effect of randomness, we first define a term called ‘early-stop trial’, which indicates an unusually low validation accuracy generated in an early pruning step that triggers a total (early) stop of the whole pruning process. The minimum observations show that in unusual cases, GRS performs better than JGRS with either less pruning time, a more compressed model, or higher accuracy. However, the average (‘avg’) observations support our general conclusions from the experiments: JGRS will typically produce more compressed models, with comparable accuracy, and faster pruning time than GRS. The results of the minimum observations are influenced by rarely-occurring early-stop trials. To mitigate detrimental effects due to randomness, one can simply execute JGRS multiple times and take the best result.

**Figure 13.**
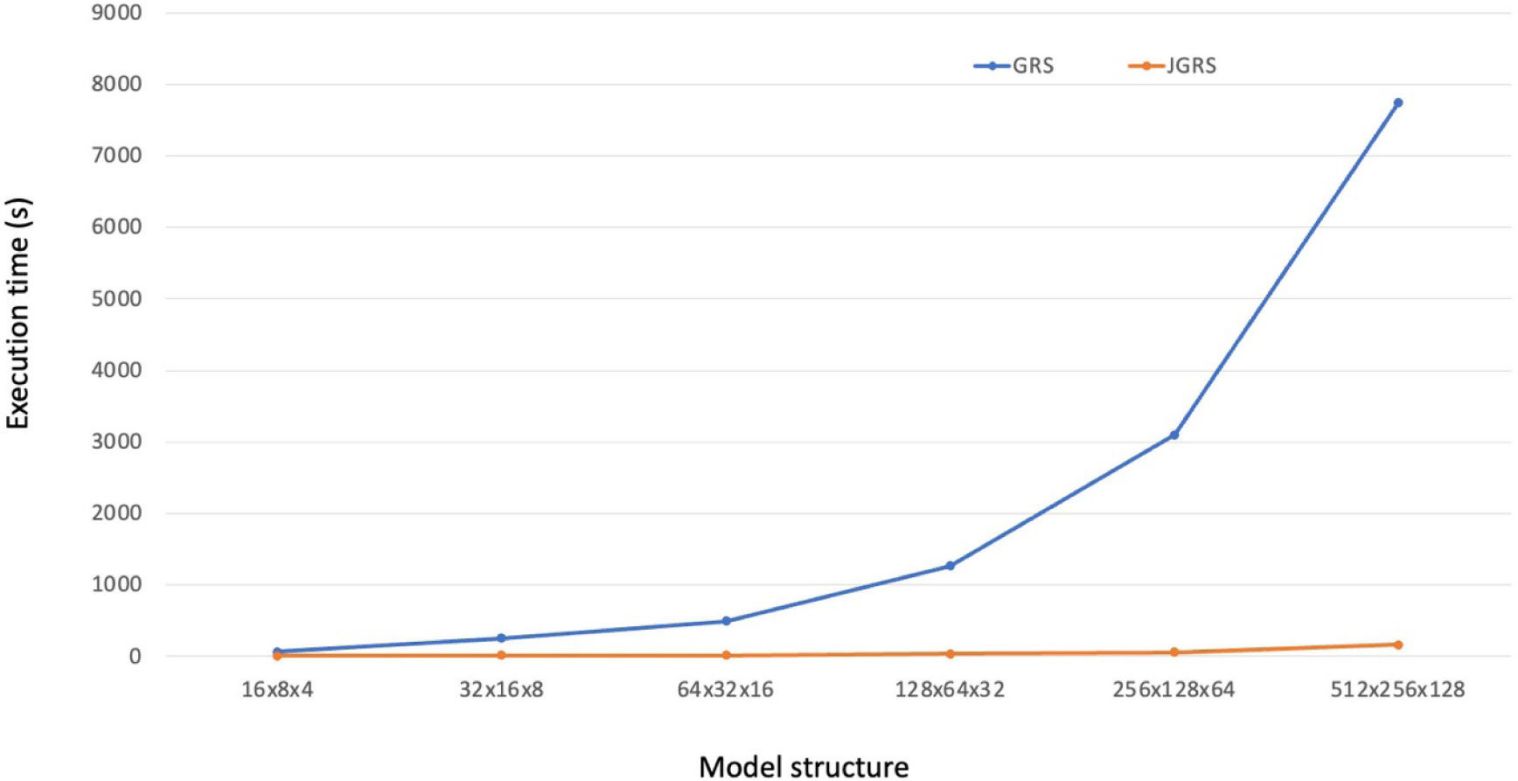
Trends of JGRS and GRS pruning speed with different sizes of MLP models on the WGEVIA-REAL dataset.

We further consider the nn1 model as a detailed case study and configure different hidden structures 16 × 8 × 4, 32 × 16 × 8, 64 × 32 × 16, 128 × 64 × 32, 256 × 128 × 64, 512 × 256 × 128 to it. We configure GRS to have 3 attempts, and JGRS to have attempt_stage_1=attempt_stage_2=attempt_stage_3=3. We apply JGRS and GRS on the WGEVIA-REAL dataset with 10 repeated trials on each initial model structure. The average runtimes of JGRS and GRS are plotted in figure 13. As the WGEVIA-REAL dataset contains well-featured embedding vectors of microcircuits, the final compact model size is small and the pruning process goes through a relatively long far phase when GRS is applied. We can see that JGRS effectively jumps across the far phase, taking a relatively small amount of time in this phase, and derives the final compact model with a small number of iterations of GRS pruning. In comparison, GRS pruning time keeps increasing with increasing model size, as GRS spends more and more time in the far phase before it reaches the final compact model.

### 3.5. JGRS for large-scale models

Since JGRS operates very efficiently when structured pruning proceeds under the far phase, JGRS can prune much larger-scale DNN models than those that can be processed by GRS using similar amounts of time and computational resources. In this section, we demonstrate the ability of JGRS to fully process large-scale input models that GRS cannot finish pruning with a reasonable time budget on the computer platform that we used for our experiments. The platform is equipped with a core i7-2600K CPU and a GeForce GTX 1080 GPU. We refer to the large-scale initial models used in these experiments as *large models*, and the previous input models (discussed in section 3.4) as *small models*. Each large model is derived by scaling up a corresponding small model. In these experiments, the accuracy drop tolerance is set as 𝒯=0.985.

Comparing the results in tables 3-5, we see that the test accuracy and validation accuracy of the pruned models resulting from large models are higher than those from the corresponding small models. The final sizes of the compact models derived from the large models are larger too. These results are because 𝒯 is the same across the experiments, and the large models generally have higher initial accuracy.

From the results in tables 3-5, we see also that the runtime of pruning large models with JGRS is shorter than pruning corresponding small models with GRS, which shows that JGRS effectively increases the pruning speed. GRS is not suitable for pruning large-scale models because GRS interleaves retraining and validation with each removal unit elimination. In contrast, JGRS implements the jump mechanism, which breaks the rapid runtime increase (in terms of the number of DNN nodes) during the far phase regime.

We also examined the pruning speed of JGRS on the WGEVIA-REAL dataset. The input models used in this experiment are the large-scale models shown in figure 3. The experiment results are averaged over ten independent trials. According to the results shown in table 6, we observe significant model size reduction through pruning within a 3% test accuracy drop. We also observe that JGRS derives much smaller compact models with faster pruning speed on the WGEVIA-REAL dataset compared to the MSN datasets. We anticipate that there are two main aspects to this more favorable performance on the WGEVIA-REAL dataset. First, the WGEVIA-REAL dataset contains more informative features that allow the JGRS pruning process to jump across a longer *far phase*. Second, the WGEVIA-REAL dataset has lower dimensionality compared to the MSN datasets, which facilitates much faster retraining.

We also conduct a comparison experiment between JGRS and the NWM method. NWM is a an abbreviation for NWM based inter-layer selection. NWM is our combined implementation of state-of-the-art weight magnitude based pruningmethods from literature—in particular, methods that are presented in [16, 18].

In our experiments, we configure NWM to prune in a layer-by-layer fashion, starting at the input side of the network and proceeding with sequential ordering from one layer to the next. We also configure NWM so that before each retraining, half of the weight parameters are removed—in particular, the weight parameters whose magnitudes are in the lower half of all magnitudes are removed. We use the same configuration for JGRS as in our other experiments. We set both JGRS and NWM to stop when the accuracy drops below the accuracy-drop tolerance 𝒯 or when a structure of the minimum allowable size is reached. We apply JGRS and NWM to prune the well-known AlexNet model structure on the WGEVIA-REAL dataset. Due to the smaller input matrix, we modify all filter widths in AlexNet to 2. Both pruning methods are repeated ten times.

According to the averaged experimental results shown in table 7, JGRS uses relatively more pruning time but generates more compact models compared to NWM. Additionally, we see from the results that JGRS is able to compress AlexNet more effectively under the same accuracy-drop tolerance.

## 4. Conclusion and discussion

In this paper, we developed new methods for scalable pruning of DNNs. These new methods have important applications in neural engineering when analysis of large collections of neurons is involved. We first analyzed GRS, which is a previously-developed state-of-the-art algorithm for DNN pruning. We performed a detailed experimental analysis of how GRS operates, and identified two distinct regimes in the operation of the algorithm. Based on insights derived from this analysis, we proposed a significantly improved structured pruning method called JGRS. JGRS builds upon the advantage of GRS, and introduces a concept called the jump mechanism, which exploits the multiple regimes of operation in GRS, and greatly improves the time-efficiency of the pruning process. Through extensive experiments on calcium-imaging-based neural signals, we demonstrated that JGRS has comparable pruning ability as GRS in terms of the accuracy of the generated compact models. At the same, we demonstrated that JGRS provides much faster pruning speed than GRS. We also demonstrated that JGRS is able to reach more compressed models compared to other weight-magnitude-based pruning methods in the literature, under same accuracy-drop tolerance.

Configurations of JGRS can be flexibly adjusted to trade off compression power and pruning time according to preferences of the system designer. For example, recall that we introduce a parameter $N_{p}$ that determines how many times JGRS can re-try in each pruning phase p if the accuracy drop tolerance is violated. The parameter, set to 2 in our experiments (for all phases), was introduced in part to reduce variations due to randomness in the results produced by JGRS. If faster pruning is prioritized (e.g. as might be desired during early-stage prototyping), then lower values of $N_{p}$ may be chosen, whereas when the degree of compression is more important, higher values of $N_{p}$ can be used. Also, it may be interesting to explore using varying $N_{p}$ values for different phases.

## Figures and Tables

**Table 1.** Compression rates in different stages of JGRS.

Algorithm stage	*CR*
Far phase 1	$\sum_{\lambda =1}^{N_{H}}midpoint(\lambda )$
Far phase 2	midpoint(λ)
Near phase	1

**Table 2.** Summary of comparison experiments of JGRS and GRS over all four input models with the MSN datasets.

JGRS vs GRS												
	AL_difference			FCI_difference			PCI_difference			Time_ratio		
Model	min	max	avg	min	max	avg	min	max	avg	min	max	avg
nn1	−8.35%	11.13%	0.28%	−45.47%	93.58%	13.05%	−45.10%	93.51%	13.03%	0.64	23.61	7.95
nn2	−11.87%	9.22%	0.65%	−40.58%	87.39%	19.75%	−40.55%	87.29%	19.72%	0.33	8.11	2.09
cnn1	−14.35%	11.65%	0.00%	−31.40%	93.04%	11.37%	−31.24%	92.98%	11.35%	0.35	18.92	5.58
cnn2	−14.46%	10.87%	0.00%	−50.23%	84.09%	8.78%	−50.20%	83.76%	8.76%	0.3	10.6	4.84

**Table 3.** Summary of pruning experiments over all four input models using JGRS on the MSN datasets.

Model	TestAcc_S(lost%)	ValAcc_S(lost%)	FLOPs_S(% of initial)	Paras_S(% of initial)	Prune_Time (s)
nn1	0.886(2.10%)	0.910(0.71%)	2410(27.80%)	1223(27.84%)	157.0
nn2	0.887(2.26%)	0.910(0.98%)	2821(36.41%)	1426(36.49%)	63.2
cnn1	0.865(2.37%)	0.877(0.67%)	3648(15.98%)	1855(16.12%)	1157.0
cnn2	0.868(2.24%)	0.892(0.71%)	3594(17.28%)	1826(17.44%)	532.2

**Table 4.** Summary of pruning experiments over all four input models using GRS on the MSN datasets.

Model	TestAcc_S(lost%)	ValAcc_S(lost%)	FLOPs_S(% of initial)	Paras_S(% of initial)	Prune_Time (s)
nn1	0.889(1.82%)	0.912(0.65%)	3801(40.86%)	1925(40.87%)	922.9
nn2	0.893(1.61%)	0.911(0.86%)	4361(56.16%)	2203(56.21%)	115.7
cnn1	0.865(2.38%)	0.88(0.46%)	7208(27.34%)	3644(27.47%)	5933.6
cnn2	0.868(2.24%)	0.893(0.55%)	5514(26.06%)	2792(26.2%)	2329.1

**Table 5.** Summary of pruning experiments over all four scaled input models using JGRS on the MSN datasets.

Model	TestAcc_S(lost%)	ValAcc_S(lost%)	FLOPs_S(% of initial)	Paras_S(% of initial)	Prune_Time (s)
nn1_scaled	0.904(1.94%)	0.927(0.73%)	5237(1.18%)	2651(1.19%)	346.8
nn2_scaled	0.904(1.99%)	0.926(0.87%)	5665(5.16%)	2863(5.17%)	138.8
cnn1_scaled	0.883(2.90%)	0.907(0.84%)	54 926(1.32%)	27 579(1.33%)	3064.9
cnn2_scaled	0.887(2.39%)	0.911(0.80%)	33 275(1.54%)	16 726(1.55%)	1016.8

**Table 6.** Summary of pruning experiments over all four scaled input models using JGRS on the WGEVIA graph embedding dataset.

Model	TestAcc_S(lost%)	ValAcc_S(lost%)	FLOPs_S(% of initial)	Paras_S(% of initial)	Prune_Time (s)
nn1_scaled	0.944(1.95%)	0.968(1.20%)	765(0.19%)	394(0.19%)	161.4
nn2_scaled	0.940(2.53%)	0.966(1.21%)	1632(1.94%)	830(1.95%)	94.6
cnn1_scaled	0.954(1.03%)	0.968(0.96%)	2841(0.08%)	1452(0.08%)	1005.5
cnn2_scaled	0.948(1.87%)	0.963(1.25%)	600(0.04%)	316(0.04%)	336.9

**Table 7.** Summary of pruning experiments over Alexnet using JGRS and NWM on the WGEVIA dataset.

Model	Pruning method	TestAcc_S(lost%)	ValAcc_S(lost%)	FLOPs_S(% of initial)	Paras_S(% of initial)	Prune_Time (s)
AlexNet	JGRS	0.946(1.77%)	0.969(0.74%)	1843(0.0%)	961(0.0%)	2488
	NWM	0.953(1.04%)	0.97(0.64%)	7419 747(10.59%)	3709 873(10.59%)	1242

## Data Availability

The data that support the findings of this study are available upon reasonable request from the authors.
